# Neuromuscular Impairment Following Backpack Load Carriage

**DOI:** 10.2478/hukin-2013-0029

**Published:** 2013-07-05

**Authors:** Sam D. Blacker, Joanne L. Fallowfield, James L.J. Bilzon, Mark E.T. Willems

**Affiliations:** 1 University of Chichester, Department of Sport and Exercise Sciences, West Sussex, UK.; 2 Institute of Naval Medicine, Alverstoke, Hampshire, UK.; 3 University of Bath, Department for Health, Bath, UK.

**Keywords:** Load Carriage, Neuromuscular Impairment, Fatigue, Exercise Induced Muscle Damage

## Abstract

Load Carriage using backpacks is an occupational task and can be a recreational pursuit. The aim of this study was to investigate the mechanisms responsible for changes in neuromuscular function of the m. quadriceps femoris following load carriage. The physiological responses of 10 male participants to voluntary and electrically stimulated isometric contractions were measured before and immediately after two hours of treadmill walking at 6.5 km•h ^−1^ during level walking with no load [LW], and level walking with load carriage (25 kg backpack) [LC]. Maximal voluntary contraction force decreased by 15 ± 11 % following LC (p=0.006), with no change following LW (p=0.292). Voluntary activation decreased after LW and LC (p=0.033) with no difference between conditions (p=0.405). Doublet contraction time decreased after both LW and LC (p=0.002), with no difference between conditions (p=0.232). There were no other changes in electrically invoked doublet parameters in either condition. The 20:50 Hz ratio did not change following LW (p=0.864) but decreased from 0.88 ± 0.04 to 0.84 ± 0.04 after LC (p=0.011) indicating reduced Ca2+ release from the sarcoplasmic reticulum during excitation contraction coupling. In conclusion, two hours of load carriage carrying a 25 kg back pack caused neuromuscular impairment through a decrease in voluntary activation (i.e. central drive) and fatigue or damage to the peripheral muscle, including impairment of the excitation contraction coupling process. This may reduce physical performance and increase the risk of musculoskeletal injury.

## Introduction

The ability to carry loads is a core requirement in many occupational settings including the military ( Knapik et al., 1996 ) and emergency services ( McLellan and Selkirk, 2004 ; Richmond et al., 2008 ). During load carriage, there are often requirements to conduct physical and/or skilled tasks, such as moving over obstacles ( Knapik et al., 1996 ), firing weapons ( Knapik et al., 1997 ), fighting fires ( Bilzon et al., 2002 ) or extracting casualties ( Richmond et al., 2008 ). Decreases in neuromuscular function have been shown to impair physical and skilled performance ( Byrne et al., 2004 ) which are likely to adversely impact upon the ability to conduct these occupational tasks.

Neuromuscular function is most accurately assessed by measuring the ability of a muscle or muscle group to produce force ( Warren et al., 1999 ). Neuromuscular impairment (i.e. a reduction in the muscles force producing capability) may be caused by a combination of fatigue and structural damage, and has been shown following prolonged running, cycling and ski skating exercise ( Millet and Lepers, 2004 ). Mechanisms of neuromuscular impairment can be assessed using voluntary and electrically stimulated contractions ( Jones, 1996 ; Shield and Zhou, 2004 ). Changes in central activation [i.e. Voluntary Activation (VA)] can be measured by superimposing an electrical stimulation during a Maximal Voluntary Contraction (MVC) ( Shield and Zhou, 2004 ). Changes to the contractile properties of muscle may be assessed by the responses to electrically stimulated contractions at rest ( Jones, 1996 ; Martin et al., 2005 ).

Clarke et al. (1955) observed decreases in force producing capability of the knee, trunk and ankle flexors and extensors and the shoulder elevators following a 12.1 km road march at 4 km•h ^−1^ carrying loads of 13, 18 and 27 kg, suggesting the presence of neuromuscular impairment. However, the authors acknowledge that there was wide variation in their measurements, which probably arose from conducting the experiment in the field and the use of a rudimentary wire tension dynamometer which measured on a discrete rather than continuous scale ( Clarke et al., 1955 ). A more recent study ( Blacker et al., 2010 ) supported these findings and showed that load carriage on level and downhill gradients causes neuromuscular impairment. However, no studies have determined whether long duration treadmill walking, or load carriage per se, is the cause of neuromuscular impairment. In addition, identifying the mechanisms responsible for neuromuscular impairment can inform intervention strategies to improve occupational performance.

Therefore, the aims of this study were to use an occupationally relevant model of load carriage to: (1) quantify the effect of carrying load (25 kg backpack) on changes in neuromuscular function of m. quadriceps femoris following two hours of treadmill walking (6.5 km•h ^−1^ ) on a level gradient; and, (2) investigate the physiological mechanisms responsible for any changes in neuromuscular function following exercise. The hypotheses were that: (1) load carriage on a level gradient causes decreases in neuromuscular function compared to no changes following walking with no load and, (2) changes in neuromuscular function following load carriage would be due to both central and peripheral mechanisms.

## Material and Methods

Ten healthy male participants (age: 30 ± 8 years; body height: 1.79 ± 0.05 m; body mass: 79.4 ± 8.3 kg) volunteered. Participants had a range of previous recreational experience of carrying load in backpacks. Ethical approval for all procedures and protocols were provided by the University Ethics Committee. All procedures were performed in accordance with the Declaration of Helsinki (2004). Participants provided written informed consent and were screened to ensure they were free from any musculoskeletal injury prior to commencing the study. Participants were instructed to avoid consumption of caffeine, sports drinks or food two hours prior to the neuromuscular function tests and refrain from any vigorous physical activity in the day prior to treadmill walking.

Body mass (Seca Model 880, Seca Ltd., Birmingham, UK) and height (Avery Berkel, Smethwick, UK) were measured whilst wearing shorts and underwear.

At least five days prior to beginning the experimental protocol, participants were familiarised with all test procedures. Participants completed three maximal voluntary isometric contractions, and all electrical stimulation procedures (described in detail below). The electrical current required to induce a maximal twitch (i.e. no further increase in twitch force with increases in current) (group mean ± SD; 830 ± 54 mA) and sub-maximal twitch (5 % MVC force) (group mean ± SD; 433 ± 65 mA) which were recorded and kept constant in all subsequent test sessions.

The study was a two way cross over randomised design, where each participant performed the following conditions at least seven days apart on a motorised treadmill (Woodway Ergo ELG 70, Cranlea & Co, Birmingham, UK): (1) two hours level walking (0 % gradient) at 6.5 km•h ^−1^ carrying no load [Level Walking (LW)]; and (2) two hours level walking (0 % gradient) at 6.5 km•h ^−1^ carrying a 25 kg backpack [Level Walking with Load Carriage (LC)]. Walking speed was kept constant between test conditions and an absolute load carried, to reflect realistic occupational requirements (e.g. military load carriage). Participants completed the series of isometric contractions before and immediately after treadmill walking.

For all isometric contractions of m. quadriceps femoris, participants were secured in a custom built chair with hip and knee at 90° flexion. Velcro straps were placed around the participant’s chest and waist to restrict movement of the upper body and hips. A cuff was placed around the participant’s ankle (proximal to the fibular notch and medial malleolus) and attached to an s-beam load cell (RS 250kg, Tedea Huntleigh, Cardiff, UK) via a steel chain at the base of the chair. The force produced from the m. quadriceps femoris was recorded on a computer at 1000 Hz using Chart 4 V4.1.2 (AD Instruments, Oxford, UK). Two custom made saline soaked electrodes (9 × 18 cm) were placed just above the patella and over the muscle belly of the m.quadriceps femoris in the proximal third part of the thigh of the non-dominant leg (determined by self report). Electrodes spanned the four muscles of the m. quadriceps femoris. The position of the electrodes was marked using permanent pen to ensure accurate placement on subsequent tests. For all electrically evoked test procedures, stimulation was provided through an electrical muscle stimulator (Model DS7A, Digitimer Limited, Welwyn Garden City, UK). Multiple pulses were controlled by a NeuroLog pulse generator (Digitimer Limited, Welwyn Garden City, UK). When participants were first attached to the chair they conducted three 5 s sub-maximal contractions (∼200 N) to become accustomed to the experimental set up.

Maximal voluntary contraction (MVC): Participants were initially instructed to relax, and then to take up the tension in the connecting chain (exerting a minimal force on the strain gauge). Participants then produced a 3 to 5 s MVC with strong verbal encouragement. Approximately 90 % of MVCs provided maximal force on the first attempt. The maximal force was taken as the single absolute highest force value during the contraction.

Interpolated doublet (% voluntary activation) during isometric contraction: participants were instructed to produce an MVC (as described above). A doublet pulse (two maximal single twitches separated by a 10 ms gap) was applied to the m. quadriceps femoris during the plateau phase of the contraction (superimposed doublet), and immediately after the MVC when participants returned to rest (post MVC doublet). Percent voluntary activation (% VA) was calculated (Equation 1). The following parameters were calculated for the post MVC doublet: (a) peak force (N), the maximal force value of the doublet; (b) contraction time (s), the time between the first derivation from baseline and peak torque; (c) average rate of force development (N•s ^−1^ ), peak force/contraction time; (d) half relaxation time (s), the time taken to fall from peak force to half of the value during the relaxation phase; (e) maximal rate of force development (N•s ^−1^ ), the highest value of the first derivative of the force signal; and (f) maximal rate of force decrease (N•s ^−1^ ), the lowest value of the first derivative of the force signal.

Isometric 20 Hz and 50 Hz stimulation: participants were instructed to relax then take up the tension in the connecting chain (exerting a minimal force on the strain gauge). 20 Hz and 50 Hz stimulations (0.5 s duration), with 30 s rest between stimulations, were applied to the m. quadriceps femoris using the sub-maximal twitch current ( Millet and Lepers, 2004 ). A reduction in the ratio of the forces at 20 Hz and 50 Hz would show the presence of low frequency fatigue (LFF), which would indicate impaired calcium release during the excitation-contraction coupling process ( Jones, 1996 ).

Environmental temperature was recorded using a dry bulb thermometer (Fisher Scientific, Loughborough, UK). No differences in environmental temperature were observed between conditions or tests (p>0.05), for LW (pre 21.4 ± 1.6 vs. post 21.4 ± 1.6°C) or LC (pre 21.5 ± 1.6 vs. post 21.5 ± 1.3 °C).

Statistical analysis was undertaken using SPSS for Windows V15 (SPSS, Chicago, Illinois) and post-hoc analysis in Microsoft Excel 2002 for Windows. Normal distribution of the data was verified using the Kolmogorov-Smirnov test. Differences between groups and over time were assessed using 2-way repeated measures ANOVA. If sphericity was violated, the Greenhouse-Geisser correction was used. Post-hoc analysis of significant main effects and interaction were conducted using the Tukey’s HSD test. Effect sizes (Cohen’s r) were calculated ( Cohen, 1988 ). The results are presented as mean ± standard deviation (SD). Statistical significance was set a priori at p<0.05.

## Results

**Figure 1:**
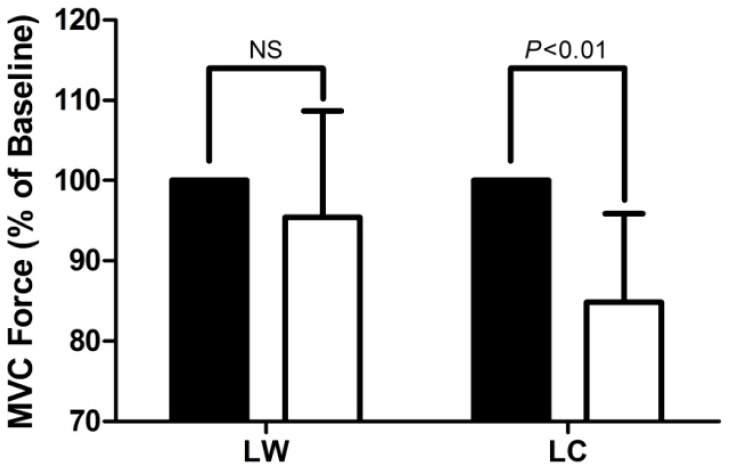
*
Force of the m. quadriceps femoris during isometric MVC, measured before (* ▪ *) and immediately after (* □ *) two hours of treadmill walking (6.5 km·h ^−1^ ) on a level gradient with no load (LW) or load carriage (25 kg backpack) (LC) (n = 10).
*

There was an interaction effect showing the change in isometric MVC force over time was different between LW and LC (p=0.009, r=0.74). Following LW, there was no change in MVC force (p=0.292, r =0.33). However, after LC MVC force decreased by 15 ± 11 % (p=0.006, r =0.53) ( Figure 1 ). VA during the MVC decreased after both LW and LC (p=0.033 r=0.64,), but there was no interaction effect (p=0.405, r=0.28) ( Table 1 ). Doublet contraction time decreased after both LW and LC (p=0.002, r=0.82) but there was no interaction effect (p=0.232, r=0.39). The pre-exercise value of the ratio of 20:50 Hz stimulations was lower for LW than LC (p=0.011, r=0.51) ( Table 1 ). There was an interaction effect in the change in the 20:50 Hz ratio over time (p=0.037, r=0.63), following LW there was no change (p=0.864, r=0.14), however, after LC the 20:50 Hz ratio decreased (p=0.011, r=0.51) ( Table 1 ). Doublet peak force, average rate of doublet tension development, doublet half relaxation time, doublet maximal rate of force development and doublet maximal rate of force decrease did not change following LW or LC ( Table 1 ).

## Discussion

This study investigated changes in neuromuscular function following two hours of load carriage during level treadmill walking with and without load. The novel approach of this study was to investigate these changes using voluntary and electrically stimulated contractions in a controlled laboratory setting. Walking unloaded for two hours at 6.5 km•h ^−1^ on a 0 % gradient did not change the force producing capability of the knee extensors but did affect some contractile properties. The addition of carrying a 25 kg backpack caused a reduction in MVC force, a change in VA and 20:50 Hz indicated that this change could be due to alterations in central nervous system control and contractile properties of the muscle. These observed contrasts yielded large effect sizes (i.e. r>0.50; Cohen, 1988 ). These findings confirm the hypothesis that load carriage on a level gradient causes decreases in neuromuscular function, and that these changes were due to both central and peripheral mechanisms.

The present study supports Clarke et al. (1955) , who showed decreases in strength of the knee extensors following a 12.1 km road march at 4 km•h ^−1^ carrying loads up to 27 kg. However, Clarke et al. (1955) acknowledged that there was large variation in their measurements, likely due to the equipment used to measure changes in strength. Unlike Clarke et al. (1955) , the present study also included an unloaded walking control condition to examine if the changes in neuromuscular function were due to walking or load carriage per se.

The addition of carrying load during treadmill walking caused a decrease in the force produced by the knee extensors. When carrying a load, additional muscle fibres are recruited to maintain posture and movement on the treadmill ( Ghori and Luckwill, 1985 ), and gait is altered causing increases in knee and femur ranges of motion ( Attwells et al., 2006 ). The additional load increases ground reaction forces, therefore increasing the amount of force that the supporting muscles must absorb ( Tilbury-Davis and Hooper, 1999 ). These changes are likely to increase mechanical damage to muscle tissue as greater force is absorbed during the eccentric component of the stretch shortening cycle of the m. quadriceps femoris, causing the neuromuscular impairment following LC.

The decrease in the force produced by the knee extensors was accompanied by a decrease in VA immediately after LC ( Table 1 ). This suggests that part of neuromuscular impairment was due to central mechanisms ( Shield and Zhou, 2004 ). The decreases in VA was less than that observed following a 30 km running race ( Millet et al., 2003 ), 300 min treadmill run at 55 % maximal aerobic velocity ( Place et al., 2004 ) and 65 km ultramarathon ( Millet et al., 2002 ). There was also a decrease in VA following LW. However, this was not sufficient to cause a reduction in MVC force. These results suggest that some psychological factors may contribute to the observed decrements in performance and physical capability during prolonged walking with and without load. Further research is required to examine the mechanisms associated with the reduced central drive to skeletal muscle following prolonged walking, and the impact this might have on subjective feelings such as fatigue, tiredness and arousal.

Comparisons between conditions for the 20:50 Hz ratio should be interpreted with caution, as the pre-exercise values were greater for LC compared with LW. Warren et al. (2002) showed in animal models that approximately 75 % of the strength loss during the first 72 h following muscle injury could be attributed to failure of the excitation-contraction coupling pathway. The decrease in the 20:50 Hz ratio following LC showed the presence of LFF ( Jones, 1996 ), which is a common occurrence after prolonged exercise ( Millet and Lepers, 2004 ). LFF would indicate a reduction in Ca2+ release from the sarcoplasmic reticulum ( Jones, 1996 ) as a consequence of damage to the structure of the muscle fibre and impairment of the excitation-contraction coupling process ( Allen et al., 2008 ). This finding confirms a peripheral component to the neuromuscular impairment following LC. During prolonged exercise, such as load carriage, this damage is most likely due to eccentric contractions and the shock wave caused by increased ground reaction forces when walking rather than metabolic changes ( Millet and Lepers, 2004 ). Reductions in muscle’s force producing capability and Ca2+ release have also been shown to be closely associated with reduced muscle glycogen concentration ( Chin and Allen, 1997 ). Whole body changes in substrate oxidation (measured indirectly by the respiratory exchange ratio) have previously been shown during two hours of load carriage ( Blacker et al., 2009 ).

Therefore, changes in glycogen availability may have contributed to the reduction in neuromuscular function, but direct measures of muscle glycogen content would need to be undertaken to confirm this hypothesis.

The change in doublet parameters also indicated disruption to the excitation-contraction coupling process and peripheral impairment of muscle fibres. The slower contraction time of the doublet immediately after LC was consistent with the observation that fatigued muscles generally show a slowing of relaxation velocity ( Allen et al., 1995 ). This adds further support to the observations of disruption to the excitation contraction coupling process, probably reflecting a reduced Ca2+ release ( Martin et al., 2005 ). Interestingly, doublet contraction time also decreased following LW, which suggested that some fatigue or damage was caused to the m. quadriceps femoris during LW. Nevertheless, this did not appear to impair its force producing capability.

Findings from the present study will be of particular importance in occupational settings where physical or skilled tasks may need to be undertaken following load carriage. The decrease in neuromuscular function measured in the present study would likely impair endurance, strength and skilled task performance, and increase the risk of musculoskeletal injury. Neuromuscular impairment has a negative impact on endurance activities, causing increases in the rate of oxygen uptake and heart rate at a set running pace ( Chen et al., 2007 ), and decreases in insulin sensitivity and increased resting metabolic rate ( Tee et al., 2007 ). Neuromuscular impairment has also been shown to decrease motor control during skilled tasks ( Byrne et al., 2004 ) and sprint performance ( Twist and Eston, 2005 ). In animal models, when muscle’s force producing capability is reduced, there is a reduction in the amount of energy a muscle can absorb, that in turn would increase the risk of muscle strain ( Mair et al., 1996 ). Muscle also provides protection to the skeleton; during impact loading, muscle acts as a shock absorber to attenuate loads as they are transmitted along the proximal chain ( Warden et al., 2006 ). When a muscle is fatigued or damaged, its ability to absorb force is impaired. This impairment would increase loading on the skeleton, therefore increasing the risk of stress fracture ( Warden et al., 2006 ). The implications of neuromuscular impairment should be considered by practitioners, physical trainers and individuals engaged in load carriage activities, particularly when planning tasks which need to be completed after load carriage. Also, for load carriage physical training programmes, adequate recovery time should be provided to minimise the risk of musculoskeletal injuries. Inadequate recovery time may predispose individuals to injury, particularly during periods of reduced neuromuscular joint control/stability. Further research should evaluate the possible impact of load carriage on more muscle groups and the performance of common occupational tasks, and identify optimal recovery times when undertaking physical training to improve load carriage performance.

In conclusion, the addition of carrying load on a level gradient caused decreases in the m. quadriceps femoris force producing capability (i.e. neuromuscular impairment). Electrically evoked isometric contractions revealed that neuromuscular impairment was due to a combination of central and peripheral mechanisms, including a decrease in voluntary activation (i.e. central drive) and disruption of the excitation contraction coupling process. This is of importance in occupational settings, as neuromuscular impairment following load carriage is likely to have a negative impact on performance of strength, endurance and motor control tasks, and may increase the risk of musculoskeletal injury.

## Figures and Tables

**Table 1:** *
Voluntary and electrically stimulated isometric contractions of the m. quadriceps femoris measured before and immediately after two hours of treadmill walking (6.5 km·h* *^−1^* *) on a level gradient with no load (LW) or load carriage (25 kg backpack) (LC) (n=10)
*

** Variable **	** Condition **	** Pre-exercise **	** Post-exercise **
MVC (N)	LW	660 ± 155	617 ± 111
	LC	692 ± 141	584 ± 126 **

VA (%)	LW	98 ± 3	96 ± 6 *
	LC	95 ± 5	91 ± 10 *

Doublet Peak Force (N)	LW	174 ± 40	167 ± 43
	LC	180 ± 41	166 ± 35

Doublet Contraction Time (s)	LW	0.186 ± 0.011	0.182 ± 0.012 *
	LC	0.191 ± 0.013	0.181 ± 0.008 *

Average Rate of Doublet Tension Development (N·s ^ −1 ^ )	LW	931 ± 175	912 ± 202
	LC	937 ± 168	916 ± 172

Doublet Half Relaxation Time (s)	LW	0.096 ± 0.010	0.099 ± 0.015
	LC	0.095 ± 0.008	0.092 ± 0.007

** Doublet Maximal Rate of Force Development (N·s ^ −1 ^ ) **	LW	1536 ± 332	1482 ± 371
	LC	1588 ± 324	1450 ± 289

Doublet Maximal Rate of Force Decrease (N·s ^ −1 ^ )	LW	−1255 ± 239	−1199 ± 347
	LC	−1308 ± 291	−1238 ± 214

20:50 Hz Ratio (n=9)	LW	0.84 ± 0.03 ^*†*^	0.84 ± 0.03
	LC	0.88 ± 0.04	0.84 ± 0.04 *

*Difference from pre-exercise measurement*

* *P<0.05*

** *P<0.01*

*Difference in pre-exercise measurement between LW and LC*

† *P<0.05*
